# Benign Share Benefit to Malignant: Balanced Mixing on Feature Space for Imbalanced Breast Cancer Classification

**DOI:** 10.3390/bioengineering13070765

**Published:** 2026-06-30

**Authors:** Farchan Hakim Raswa, Muhammad Fadlurrohman, Bach-Tung Pham, Ika Candradewi, Afiahayati, Ming-Hsiang Su, Chung-I Huang, Kuo-Chen Li, Shih-Lun Chen, Yung-Hui Li, Jia-Ching Wang

**Affiliations:** 1 Department of Computer Science and Information Engineering, National Central University, Taoyuan 320317, Taiwan; farchan.hakim.r@g.ncu.edu.tw (F.H.R.); muhammadfadlurrohman@mail.ugm.ac.id (M.F.); loptruong1th1@gmail.com (B.-T.P.); jcw@csie.ncu.edu.tw (J.-C.W.); 2 Department of Computer Science and Electronics, Universitas Gadjah Mada, Yogyakarta 55281, Indonesia; ika.candradewi@ugm.ac.id (I.C.); afia@ugm.ac.id (A.); 3 Department of Data Science, Soochow University, Taipei 100006, Taiwan; 4 Department of Management Information Systems, National Chung Hsing University, Taichung 402202, Taiwan; schumi@nchu.edu.tw; 5 Department of Information Management, Chung Yuan Christian University, Taoyuan 320314, Taiwan; 6 Department of Electronic Engineering, Chung Yuan Christian University, Taoyuan 320314, Taiwan; chrischen@cycu.edu.tw; 7 Research Center for Semiconductor Materials and Advanced Optics, Chung Yuan Christian University, Taoyuan 320314, Taiwan; 8 AI Research Center, Hon Hai Research Institute, Taipei 114065, Taiwan

**Keywords:** mammography, class imbalance, feature-space augmentation, breast cancer classification, deep learning

## Abstract

A deep learning model with an imbalanced mammography dataset can bias models toward common benign BI-RADS categories and reduce recognition of less frequent malignant or high-risk categories. To address this issue, we propose B2M (Benign Share Benefit to Malignant), a model-agnostic framework for imbalance-aware multi-class BI-RADS classification in C-View mammography. B2M uses a two-phase training strategy that combines dual sampling with feature-space mixing. In Phase I, the model is trained with dual sampling, integrating instance-based and class-balanced sampling to increase minority-class representation while preserving majority-class diversity. In Phase II, the model is fine-tuned with feature-space mixing using samples from the two sampling streams. A soft-target regularization objective supervises the mixed features using labels from both streams, encouraging smoother decision boundaries across BI-RADS categories. We evaluated B2M on an imbalanced mammography cohort from the C-View EMBED dataset using stratified 5-fold cross-validation across multiple CNN backbones. C-View is a synthesized 2D mammographic image generated from 3D digital breast tomosynthesis data, capturing DBT-derived structural information while requiring less memory and computation than processing the full 3D image volume. Among these experiments, ResNeXt-50 with B2M achieved the highest balanced accuracy and Macro-F1 scores compared with the evaluated oversampling and mixing-based methods. This improvement requires an offline training-time overhead of approximately 2.81×, but it does not increase inference cost. Overall, the results suggest that B2M may be useful for imbalanced multi-class BI-RADS classification in C-View mammography. However, the findings are based on the EMBED cohort, and further validation, including external and prospective evaluation, is needed before clinical use.

## 1. Introduction

Breast cancer remains one of the most prevalent malignancies worldwide and a leading cause of cancer-related mortality among women. Early detection is crucial because treatment outcomes are strongly associated with stage at diagnosis. Mammography is the primary X-ray imaging modality for population-level screening and clinical assessment of breast abnormalities, including masses, asymmetric density, suspicious calcifications, architectural distortion, tissue retraction, and skin thickening. In routine practice, lesions are broadly categorized as benign (non-cancerous) or malignant (cancerous), and this benign-versus-malignant distinction directly affects follow-up strategy and intervention planning.

In recent years, deep learning-based computer-aided diagnosis has been widely investigated across diverse medical imaging tasks, including CT image analysis [1,2], cell nuclei classification [3], histopathology image analysis [4], and mammogram interpretation [5,6]. Compared with traditional handcrafted-feature pipelines, convolutional neural networks (CNNs) automatically learn multi-level image features from large annotated datasets, reducing the need for manually designed descriptors. This ability is useful in mammography screening, where models must detect subtle lesions, tissue asymmetries, and density patterns across large image volumes. In breast imaging, clinical practice is increasingly incorporating 3D digital breast tomosynthesis (DBT) alongside conventional 2D full-field digital mammography (FFDM). Compared with FFDM, DBT provides volumetric slice information that reduces tissue overlap and improves the visualization of subtle breast abnormalities; however, the resulting volume data increase computational and storage demands. Synthetic 2D mammography (C-View) addresses this limitation by compressing DBT information into a single projection image while enhancing high-contrast findings, such as microcalcifications and architectural distortions [7]. Therefore, deep learning models for C-View mammography must capture these enhanced tissue patterns while preserving clinically relevant local structural information [8,9].

**Figure 1:**
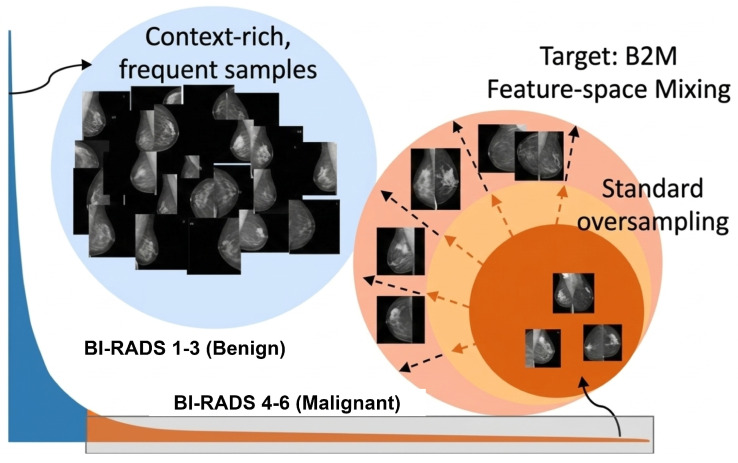
Illustration of the proposed B2M framework under severe class imbalance. Unlike conventional oversampling methods that replicate or locally interpolate rare malignant samples within a limited feature region, B2M transfers diverse structural information from well-represented, context-rich benign samples to enrich the malignant feature space.

Despite this progress, real-world medical datasets are strongly imbalanced, where benign samples (majority class) are substantially more abundant than malignant samples (minority class) (see Figure 1). In clinical practice, this challenge extends beyond a simple binary distinction. Breast cancer screening spans multiple BI-RADS categories, with a highly non-uniform class distribution. Actionable high-risk categories, such as BI-RADS 5 and 6, are severely underrepresented compared with screening-dominant benign categories, such as BI-RADS 1 and 2. In benign-versus-malignant mammography classification, this imbalance biases training toward the majority class and weakens minority-class recognition [6,10]. If this skewed distribution is handled suboptimally, model predictions may compromise clinical decision making by increasing false negatives, which may delay the detection of malignant findings, or by increasing false positives, which may lead to unnecessary follow-up procedures and patient anxiety. Therefore, effective imbalance mitigation is essential for reliable clinical deployment.

Existing imbalance-learning methods can be broadly categorized into data-level and objective-level strategies. Data-level methods increase minority-class representation by generating additional samples through synthetic augmentation [11,12]. Objective-level methods, such as focal loss [13], modify the loss function so that minority or difficult samples contribute more strongly during optimization. Despite their benefits, both strategies have limitations in clinical mammography workflows. Synthetic generation typically requires an additional generative model, such as a GAN or diffusion model, and its outputs must be carefully controlled to avoid artificial textures that could obscure microcalcifications, lesion margins, or subtle architectural distortions. Loss re-weighting also has limitations under severe class imbalance. When rare classes receive large loss weights, the model may become overly sensitive to a small number of minority samples, including noisy or ambiguous cases. This can improve minority recall but may also reduce specificity and increase false-positive predictions [14,15]. Another line of research focuses on oversampling and sample mixing. CMO [16] and Balanced-MixUp [17], for example, create synthetic training samples by mixing majority- and minority-class images in the input space. This strategy is efficient at inference time because it does not require an additional model during deployment, increases the diversity of minority-class feature information during training, and can be integrated with different CNN-based classifiers [18]. However, for mammography, image-space interpolation can be affected by acquisition noise, contrast differences, and anatomical misalignment. As a result, the mixed image may not preserve lesion-level semantic consistency, particularly for small or low-contrast findings.

Motivated by these observations, we propose B2M (**Benign Share Benefit to Malignant**), a feature-space mixing framework for imbalanced breast cancer classification. Rather than mixing at the image level, B2M performs interpolation in the latent representation space, which encodes mammography-specific semantic information and is less sensitive to image artifacts and noise. The main technical contribution of B2M is the integration of dual sampling, feature-space mixing, and soft-target regularization into a two-phase training strategy designed for the highly skewed multi-class distribution in mammography. The contributions are summarized as follows:

- We introduce a two-phase training strategy for imbalanced breast cancer classification. Because feature-space mixing is more reliable when the encoder has already learned preliminary data-specific representations, Phase I first uses dual sampling to establish a stable latent space, and Phase II then fine-tunes the model with feature-space mixing and soft-target regularization.
- In Phase I, the model is trained using a dual sampling strategy that is conceptually inspired by Balanced-MixUp [17] and adapted to the multi-class BI-RADS setting. This strategy combines instance-based and class-balanced sampling to improve minority-class exposure while preserving informative majority-class diversity.
- In Phase II, B2M transfers benign-class information to malignant classes through feature-space mixing. Building on Manifold Mixup [19], we apply latent linear interpolation to this highly imbalanced clinical task. By moving interpolation from image space to deeper representation layers, B2M operates on semantically richer features, reduces sensitivity to pixel-level artifacts, and stabilizes the latent manifold under severe class imbalance [20]. Soft-target regularization, following the principle of MixUp [21], then supervises the mixed features using labels from the two sampling streams.

Moreover, B2M does not add inference-time computational overhead, although it increases offline training cost because feature-space mixing is applied during fine-tuning. It can be integrated into end-to-end deep learning pipelines without requiring an external generator. We evaluated the proposed framework on EMBED C-View mammography images, demonstrating its applicability to imbalanced multi-class BI-RADS classification in a large clinical dataset.

## 2. Related Work

### 2.1. Deep Learning on Mammography, C-View, and the EMBED Benchmark

Automated mammography classification has advanced rapidly with deep learning. Compared with traditional radiomics pipelines, deep models learn discriminative patterns directly from X-ray images and reduce dependence on handcrafted features. Early studies mainly adopted CNN-based architectures to automate feature extraction and improve diagnostic consistency. For example, ref. [22] proposed a hybrid CNN-LSTM model that captures spatial features with convolutional layers and inter-view dependencies with Long Short-Term Memory units. Similarly, ref. [10] proposed a class-balanced CNN framework to reduce false-negative rates under class imbalance, while ref. [23] compared modern CNN and Transformer models and reported that advanced convolutional designs, such as ConvNeXT, remain effective for local tissue-pattern analysis in mammography. To model long-range context, recent studies explored Vision Transformers (ViTs) and attention-based architectures. MV-Swin-T [24] was introduced to model spatial relationships across multi-view mammograms, whereas MIME-ViT [25] was designed to process mammograms as patch sequences for capturing multi-scale morphology. To bridge these paradigms, ref. [26] proposed an attention-based hybrid framework for multi-scale and multi-view fusion, and TransBreastNet [27] was introduced as a unified CNN–Transformer model that combines image features with temporal clinical indicators to predict lesion progression and cancer subtypes.

In recent years, computer-assisted diagnosis in breast imaging has increasingly incorporated 3D digital breast tomosynthesis (DBT) in addition to conventional 2D full-field digital mammography (FFDM). Compared with FFDM, DBT provides volumetric slice information that reduces tissue overlap and improves the visualization of subtle breast abnormalities; however, this richer representation also increases computational and storage demands. Synthetic 2D mammography (C-View) addresses this challenge by compressing 3D DBT information into a single projection image while enhancing high-contrast findings such as microcalcifications and architectural distortions [28]. Therefore, deep learning models developed for C-View images must capture these enhanced tissue patterns while preserving clinically relevant local structural information [8]. Model validation in this setting requires large, clinically representative datasets. The EMory BrEast imaging Dataset (EMBED) provides a large-scale multi-view mammography resource for this purpose [29]. Recent EMBED-based benchmarks highlight the difficulty of automated Breast Imaging-Reporting and Data System (BI-RADS) categorization under realistic screening conditions [30,31]. In particular, classifiers must distinguish common benign categories (BI-RADS 1–3) from much rarer malignant or high-risk categories (BI-RADS 4–6). Under this long-tailed distribution, standard cross-entropy training can become biased toward the majority classes, reducing sensitivity to clinically important minority categories [31]. This limitation motivates the development of an imbalance-aware feature learning framework for C-View mammography images.

### 2.2. Dealing with Data Imbalance

**Re-weighting Methods.** Re-weighting addresses class imbalance by assigning different importance to training samples at the class level or instance level. At the class level, inverse class frequency [32,33] scales loss by class counts. Class-balanced loss [34] uses the effective number of samples. LDAM loss [35] enforces larger margins for minority classes. Balanced Softmax [36] adjusts decision boundaries using class priors. LADE loss [37] disentangles label distributions under distribution shift. At the instance level, focal loss [13] down-weights easy examples and emphasizes hard samples. Influence-balanced loss [38] controls each instance’s contribution to parameter updates. However, re-weighting can overfit minority-class noise and outliers because large weights may amplify unrepresentative samples [18].

**Oversampling and Generative Methods.** Re-sampling methods modify the training distribution to mitigate class imbalance [39]. Undersampling removes majority-class samples, but may discard useful information and often fails under severe imbalance. In contrast, Random Oversampling (ROS) [40] duplicates minority-class samples until classes are balanced, but this can increase overfitting risk [41]. To reduce this issue, the Synthetic Minority Over-sampling Technique (SMOTE) [42] generates new samples by interpolating minority examples. In mammography, integrated SMOTE-based approaches [43] were proposed to improve classification on imbalanced datasets. For generative augmentation, GANs [44] synthesize realistic minority-class data. For example, a prior-guided GAN [11] generates high-fidelity mammograms using anatomical priors such as breast density and tumor location. Similarly, SNGAN [12] was proposed to augment mammography mass classification in low-data settings. However, these generative methods generally require high computational cost and large-scale datasets to produce stable and reliable synthetic samples.

### 2.3. Augmentation and Mixup Methods

Image-space augmentation is widely used in computer vision. Cutout [45] removes random image regions, while CutMix [46] replaces masked regions with patches from another sample. Mixup [21] linearly interpolates image pairs to improve generalization and calibration. Because augmentation is closely related to oversampling, several imbalance-learning methods incorporate Mixup variants. Balanced-Mixup [17] interpolates between instance-level (imbalanced) and class-level (balanced) samples. Context-rich Minority Oversampling (CMO) [16] applies CutMix to combine minority foreground with majority background.

Despite these advances, feature-space augmentation for imbalanced medical classification remains underexplored. Prior studies [19,20] suggest that feature-space interpolation is more semantically meaningful and less sensitive to pixel artifacts than image-space interpolation. This property is especially relevant in mammography, where acquisition noise and scanner variability can degrade pixel-space mixing quality [47,48]. Therefore, feature-space interpolation offers a promising direction for improving minority-class representations while maintaining stable optimization.

### 2.4. Position with Other Studies

As summarized in Table 1, B2M is closely related to Balanced-MixUp [17], CMO [16], and standard MixUp [21]. Similar to these methods, B2M uses soft-target supervision to regularize training under class imbalance. B2M differs primarily by applying the mixing operation in latent feature space rather than directly in the input image space. This design preserves mammography-specific semantic information, where subtle structures such as microcalcifications, masses, and architectural distortions can be sensitive to pixel-level interpolation.

The two-phase strategy also separates representation learning from feature-space fine-tuning. In Phase I, dual sampling helps the model learn a stable representation. In Phase II, feature-space mixing with soft-target regularization refines this representation. Unlike static re-weighting methods [13,34,35], B2M adds representation-level regularization. Unlike GAN-based augmentation [11,12], it does not require a separate generative model. The trade-off is an additional offline training cost during fine-tuning.

## 3. Proposed Method: Benign Share Benefit to Malignant (B2M)

### 3.1. Overview

**Figure 2:**
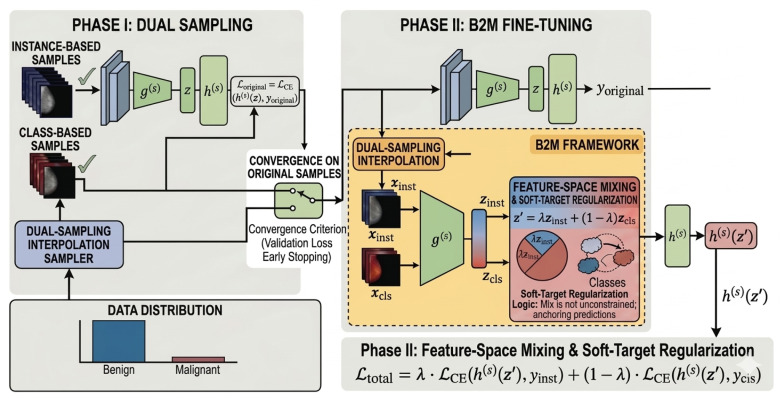
Overview of the proposed B2M framework. Phase I uses dual sampling to learn a stable feature space from instance-based and class-balanced streams. Phase II interpolates latent features from the two streams and supervises the mixed prediction with a soft label derived from $y_{\mathrm{inst}}$ and $y_{\mathrm{cls}}$. We note that the binary benign/malignant illustration is used for visual simplicity. The proposed design therefore targets imbalanced multi-class BI-RADS classification.

We propose a framework that combines class-aware sampling with feature-space Mixup for imbalanced mammography classification. B2M is implemented as a two-phase training strategy: Phase I trains the model with dual sampling, and Phase II fine-tunes the model with feature-space mixing and soft-target regularization. Let x∈X denote an input image and y∈{1,…,C} denote its class label across *C* categories. For loss computation and feature mixing, each class label is represented as a one-hot target vector. We define $g^{\left(s\right)}\left(\cdot \right)$ as the mapping function of a CNN encoder up to stage *s*, such that $z=g^{\left(s\right)}\left(x\right)$ is the latent feature at that stage. We generate a synthetic mixed representation $z^{\prime }$ by interpolating latent layers from an instance-based sampling stream and a class-balanced sampling stream, denoted as $g^{\left(s\right)}\left(x_{\mathrm{inst}}\right)$ and $g^{\left(s\right)}\left(x_{\mathrm{cls}}\right)$ respectively. During Phase II, soft-target regularization is applied to keep predictions on mixed samples consistent with minority-class semantics. The complete pipeline of our proposed B2M training strategy is depicted in Figure 2.

### 3.2. Re-Weighting Class Distribution

In highly imbalanced datasets, optimization is often dominated by majority-class samples, which weakens minority-class learning. In Phase I, we train the model using a dual sampling strategy that combines instance-based sampling with class-balanced sampling. This design increases minority-class representation while preserving the natural data distribution.

For a training set $D={\left\{\left(x_{i},y_{i}\right)\right\}}_{i=1}^{N}$ with *C* classes, let $n_{j}$ denote the number of samples in class *j*. The sampling probability for class j∈{1,…,C} is defined as:

(1) $$p_{j}=\frac{n_{j}^{q}}{\sum_{k=1}^{C}n_{k}^{q}}$$

The hyperparameter q∈[0,1] controls the balance-strength trade-off. When q=1, sampling follows the original class frequency (instance-based sampling). When q=0, sampling becomes uniform ($p_{j}=1/C$), corresponding to class-balanced sampling. Intermediate values (0<q<1) provide a smooth interpolation between these two regimes, enabling controlled re-balancing without discarding informative majority-class samples. In this framework, Phase I optimization explicitly utilizes dual sampling streams operating in parallel: an instance-based stream setting q=1 to capture structural density, and a class-balanced stream setting q=0 to ensure uniform minority exposure.

### 3.3. Feature-Space Mixing

In Phase II, after the model has learned an initial representation from dual sampling, we fine-tune it using Mixup in latent feature space rather than in input space. Feature-space interpolation operates on higher-level semantic representations and is less sensitive to pixel-level artifacts, which is beneficial for medical images with heterogeneous acquisition conditions. Let the encoder be decomposed at stage *s* as:

(2) $$f\left(x\right)=h^{\left(s\right)}\left(g^{\left(s\right)}\left(x\right)\right)$$

where $g^{\left(s\right)}\left(\cdot \right)$ maps the input to stage-*s* features and $h^{\left(s\right)}\left(\cdot \right)$ maps these features to the final prediction. Given a sample $\left(x_{\mathrm{inst}},y_{\mathrm{inst}}\right)$ from the instance-based sampling and a sample $\left(x_{\mathrm{cls}},y_{\mathrm{cls}}\right)$ from the class-balanced sampling, we construct synthetic mixed feature representations as:

(3) $$z^{\prime }=\lambda g^{\left(s\right)}\left(x_{\mathrm{inst}}\right)+\left(1-\lambda \right)g^{\left(s\right)}\left(x_{\mathrm{cls}}\right)$$

with mixing coefficient λ∼Beta(α,α). This design increases minority-class participation during training and prepares the model to regularize the decision boundary with semantically meaningful interpolants. As a result, the model improves class balance while maintaining stable optimization in the imbalanced setting.

### 3.4. Soft-Target Regularization

Feature-space mixing interpolates representations between benign and malignant classes. Without appropriate supervision, however, the resulting latent features may be difficult to train. Following the soft-target principle of MixUp [21], we assign each mixed feature a soft label formed from the labels of the two sampling streams. Let $h^{\left(s\right)}\left(z^{\prime }\right)$ denote the raw logits produced by the classification head for the mixed embedding $z^{\prime }$, and let

(4) $$y^{\prime }=\lambda y_{\mathrm{inst}}+\left(1-\lambda \right)y_{\mathrm{cls}}.$$

The training objective is then written as:

(5) $$L_{\mathrm{total}}=L_{\mathrm{CE}}\left(h^{\left(s\right)}\left(z^{\prime }\right),y^{\prime }\right)$$

where $L_{\mathrm{CE}}$ denotes cross-entropy computed from the raw logits and the soft target $y^{\prime }$. The proposed method applies soft-target supervision to feature-space samples formed from the instance-based and class-balanced sampling streams. This encourages smoother decision boundaries while keeping the supervision consistent with the mixed latent representation.

### 3.5. Training Strategy

We implement B2M as a two-phase training strategy, as summarized in Algorithm 1. In Phase I (Dual Sampling), the model is trained only with dual sampling using the class re-weighting distribution described in Section 3.2. This phase allows the encoder to learn stable mammographic representations before any synthetic feature interpolation is introduced. Applying feature-space mixing too early may generate noisy latent samples because the representation space has not yet captured reliable diagnostic structure.
**Algorithm 1** B2M Training Strategy**Require:** Training set D, Candidate layers S, Mixup hyperparameter α, Sampling indicator *q*.**Ensure:** Optimized CNN encoder $g^{\left(s\right)}$ and classifier $h^{\left(s\right)}$.
  1:**// Phase I: Dual Sampling**  2:**while** Validation loss $V_{t}$ improves within patience *n* **do**  3:   Sample $x_{\mathrm{inst}}$ via $p_{j}\left(q=1\right)$ and $x_{\mathrm{cls}}$ via $p_{j}\left(q=0\right)$;  4:   Compute $L_{\mathrm{CE}}$ on original samples and update parameters;  5:**end while**  6:**// Phase II: B2M Fine-tuning**  7:**for** each epoch $e\in \{1,\ldots ,E_{\mathrm{fine}}\}$ **do**  8:   Sample $x_{\mathrm{inst}}$ via $p_{j}\left(q=1\right)$ and $x_{\mathrm{cls}}$ via $p_{j}\left(q=0\right)$;  9:   Select manifold layer s∼S and interpolation weight λ∼Beta(α,α);10:   **// Latent Feature Space Mixing**11:   $z^{\prime }=\lambda g^{\left(s\right)}\left(x_{\mathrm{inst}}\right)+\left(1-\lambda \right)g^{\left(s\right)}\left(x_{\mathrm{cls}}\right)$;12:   **// Forward Pass on Mixed Embedding**13:   Compute predicted raw logits $h^{\left(s\right)}\left(z^{\prime }\right)$;14:   **// soft-target regularization**15:   $y^{\prime }=\lambda y_{\mathrm{inst}}+\left(1-\lambda \right)y_{\mathrm{cls}}$;16:   $L_{\mathrm{total}}=L_{\mathrm{CE}}\left(h^{\left(s\right)}\left(z^{\prime }\right),y^{\prime }\right)$;17:   Update model parameters via backpropagation $\left(\nabla_{g,h}L_{\mathrm{total}}\right)$;18:**end for**


After the model reaches initial convergence according to the validation-loss early stopping criterion, we begin Phase II (B2M Fine-tuning). Specifically, if the validation loss $V_{t}$ does not improve by a threshold ϵ for *n* consecutive epochs, Phase I is stopped and Phase II is activated. During Phase II, feature-space mixing is performed for a total of $E_{\mathrm{fine}}$ epochs using samples from both the instance-based and class-balanced sampling streams, combined with soft-target regularization. By applying feature mixing only after the encoder has stabilized, B2M refines the malignant-class decision boundary while keeping mixed-sample predictions semantically grounded to avoid semantic drift. All corresponding operational thresholds, constraints, and optimization parameters are consolidated in Table 2.

### 3.6. Model Architectures

To assess the robustness of B2M across different model capacities, we evaluated four CNN backbones with complementary architectural properties:

- **MobileNet-V2** is a lightweight architecture based on inverted residual blocks and linear bottlenecks, which improve feature transmission while preserving low-dimensional semantic information [49]. Its compact design helps reduce overfitting on limited medical datasets, and its bottlenecked feature maps provide B2M with dense latent representations for capturing subtle findings such as microcalcifications.
- **VGG-19** consists of 16 convolutional layers and 3 fully connected layers, using repeated 3×3 filters to form a simple hierarchical feature extractor [50]. This sequential structure provides stable mid-to-late representations for B2M, with features sampled from conv2 through conv4 to capture architectural distortions and asymmetric tissue patterns.
- **ResNeXt-50** extends residual learning by introducing cardinality, i.e., multiple parallel transformations within each block [51]. This split–transform–merge design captures tissue patterns through multiple receptive pathways, supporting B2M feature sampling across layer2 through layer4 for multi-scale cues such as spiculated margins and parenchymal densities.
- **DenseNet-121** connects each layer to all subsequent layers, resulting in L(L+1)/2 direct connections across *L* layers [52]. This dense connectivity improves gradient flow and feature reuse, allowing low-level edge and boundary information to remain available in deeper representations. B2M therefore samples from denseblock2 through denseblock4 to exploit this rich feature pool.

## 4. Implementation Details

### 4.1. Dataset and Clinical Definitions

**Figure 3:**
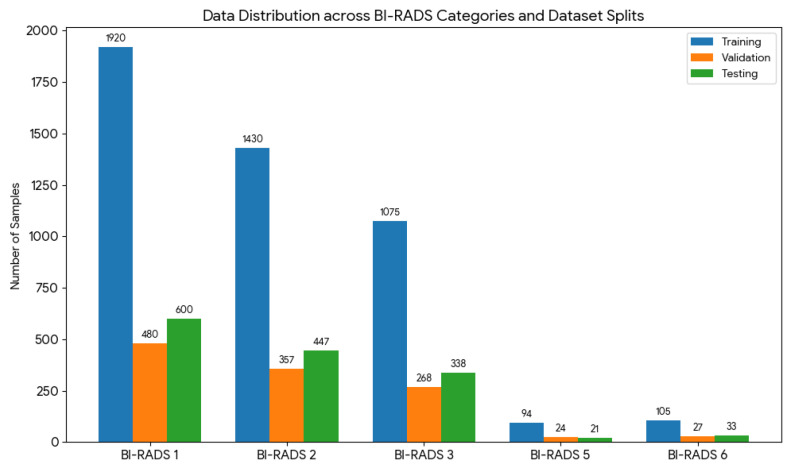
Class-imbalance distribution in the C-view EMBED cohort. Note that BI-RADS 4 is absent from the EMBED C-view cohort and is therefore not included.

We used the C-view cohort from the Emory Breast Imaging Dataset (EMBED), a large-scale clinical repository hosted on the AWS Open Data platform (https://github.com/Emory-HITI/EMBED_Open_Data, accessed on 1 January 2024) [29]. This cohort contains high-resolution C-View mammograms reconstructed from digital breast tomosynthesis (DBT), which improve lesion conspicuity and reduce tissue superimposition. The final dataset included 7219 mammograms across BI-RADS 1, 2, 3, 5, and 6. BI-RADS 4 was absent from the EMBED C-view subset and was therefore excluded from our study (as shown in Figure 3). This retained the class imbalance typical of clinical mammography screening. BI-RADS assessment categories provide a standardized clinical language for mammographic findings [53]. The included categories are defined as follows:

- **BI-RADS 1 (Negative):** No structural abnormalities or suspicious findings have been identified.
- **BI-RADS 2 (Benign):** Definitive benign findings are present, such as stable secretory calcifications, simple cysts, or fat-containing lesions.
- **BI-RADS 3 (Probably Benign):** Findings have a high probability of being benign (≤2% risk of malignancy) but warrant short-interval follow-up.
- **BI-RADS 5 (Highly Suggestive of Malignancy):** Findings have a very high probability of cancer (≥95%), necessitating appropriate clinical action.
- **BI-RADS 6 (Known Biopsy-Proven Malignancy):** Findings have been previously confirmed as malignant through pathology.

**Figure 4:**
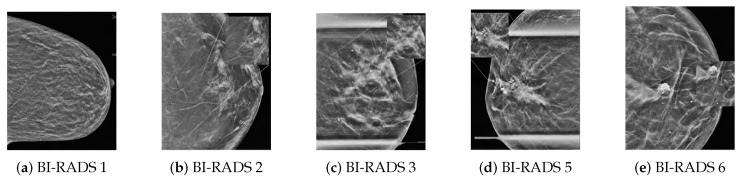
Representative C-View mammograms from the EMBED dataset across the studied BI-RADS assessment categories. Note that BI-RADS 4 is absent from the EMBED C-view cohort and is therefore not included.

Our study design defined a clinically realistic evaluation setting by retaining standard craniocaudal (CC) and mediolateral oblique (MLO) views from both breasts while accounting for view selection and laterality. Duplicate records and repeated study dates from the same examination were removed. To prevent data leakage, all data splits were performed at the patient level. Thus, images from the same patient were assigned to only one subset, regardless of view, laterality, or examination date. In the stratified five-fold cross-validation, a patient could not appear simultaneously in the training, validation, or testing subsets, ensuring that model evaluation was based on unseen patients rather than repeated images from the same individual. The representative examples are shown in Figure 4.

### 4.2. Image Preprocessing

To prepare high-resolution DICOM mammograms for deep learning, we applied a standardized preprocessing pipeline. Raw DICOM pixel intensities were first min–max normalized to the [0,255] grayscale range to reduce scanner-dependent intensity variation, and the images were then converted to lossless PNG format for efficient loading while preserving diagnostic content.

A contour-based cropping procedure was used to isolate the breast region and remove text labels, background areas, and other non-anatomical artifacts. To improve robustness across imaging systems and exposure settings, binarization was performed after intensity normalization. Each normalized image was converted to grayscale and thresholded at 50. The largest connected component was then selected as the breast region, and its bounding box was computed. All segmented images were manually audited to avoid tissue truncation caused by artifacts or low-contrast boundaries, with fewer than 0.5% of cases requiring automated boundary clamping. To obtain a square region of interest (ROI) without altering the anatomical aspect ratio, the shorter side of the bounding box was symmetrically padded to match the longer side. The square ROI was then expanded to 1.2× its side length to include peripheral contextual information. When the expanded ROI exceeded the image boundaries, the coordinates were clamped to the original image dimensions, so that only background padding, rather than breast tissue, was affected.

**Figure 5:**
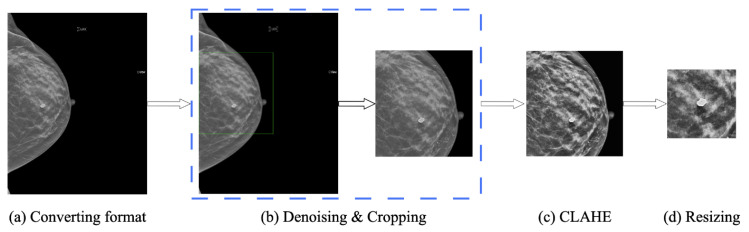
Overview of the image preprocessing pipeline, including breast region segmentation, CLAHE enhancement, and resizing.

Subtle findings, including microcalcifications and masses, were enhanced using Contrast Limited Adaptive Histogram Equalization (CLAHE) [54]. The CLAHE parameters were selected empirically through grid-search sensitivity analysis, using a clip limit of 2.0 and an 8×8 tile grid. This conservative setting limits noise amplification while improving the visibility of localized lesions. In the proposed feature-mixing framework, local contrast enhancement also helps produce clearer semantic boundaries in the latent space, reducing the likelihood that interpolated features are dominated by background artifacts. Finally, all images were center-cropped and resized to 512×512 pixels, balancing the preservation of fine diagnostic detail with computational efficiency. The complete preprocessing workflow is shown in Figure 5.

### 4.3. Evaluation Method

For imbalanced multi-class BI-RADS classification, evaluation should reflect performance across all categories rather than only the dominant benign classes. We therefore report class-balanced metrics that treat each BI-RADS category equally when aggregating performance.

(6) $$\mathrm{Balanced}\;\mathrm{Accuracy}=\frac{1}{C}\sum_{c=1}^{C}{\mathrm{Recall}}_{c}=\frac{1}{C}\sum_{c=1}^{C}\frac{{\mathrm{TP}}_{c}}{{\mathrm{TP}}_{c}+{\mathrm{FN}}_{c}}$$

(7) $$\text{Macro-F1}=\frac{1}{C}\sum_{c=1}^{C}{F1}_{c}=\frac{1}{C}\sum_{c=1}^{C}\frac{2P_{c}R_{c}}{P_{c}+R_{c}}$$

where *C* denotes the number of BI-RADS categories, and $P_{c}$ and $R_{c}$ denote precision and recall for class *c*, respectively.

### 4.4. Training and Validation Protocol

We evaluated B2M using four representative CNN backbones: MobileNet-V2 [49], VGG-19 [50], DenseNet-121 [52], and ResNeXt-50 [51]. All models were initialized with ImageNet-pretrained weights. Because these backbones require three-channel inputs, each single-channel grayscale mammogram was replicated across three channels to form a pseudo-RGB image. Geometric and photometric augmentations were applied only to the training folds, while validation and test folds remained unchanged for unbiased evaluation. To account for variation in breast positioning, ROI size, and acquisition alignment, the augmentation strategy included mild rotations up to $\pm 15^{\circ }$, scaling by 1.2×, and horizontal translations of 5%. Horizontal flips were used to increase spatial diversity and account for breast laterality. Photometric adjustments were limited to brightness and contrast changes within ±10% to simulate scanner-related intensity variability across clinical centers. Models were fine-tuned on 512×512 images using stochastic gradient descent with a learning rate of η=0.01, momentum of 0.9, and a batch size of 8. To address class imbalance, we used stratified 5-fold cross-validation, which preserves class proportions across folds. Performance was reported as the median and interquartile interval (Q1–Q3) across folds to provide a robust estimate of diagnostic generalization under imbalanced class distributions.

All remaining pipeline hyperparameters, training settings, stopping criteria, and architectural layer-selection policies (S) are summarized in Table 2. The structural block index set S denotes the backbone-specific feature stages used for latent feature mixing: conv2–conv4 for VGG-19, layer2–layer4 for ResNeXt-50, denseblock2–denseblock4 for DenseNet-121, and the corresponding inverted residual block sequences for MobileNet-V2.

## 5. Results and Discussion

We evaluate B2M on the imbalanced EMBED C-view cohort using stratified 5-fold cross-validation and report the median and interquartile interval (Q1–Q3) across folds. Because malignant prevalence is low in screening practice, plain accuracy is not an informative endpoint. We therefore prioritize *balanced accuracy* and *Macro-F1*, which jointly capture sensitivity to the minority class and class-wise precision–recall balance.

### 5.1. Comparative Analysis Across Architectures

For consistency across baseline comparisons, Table 3 reports all methods using the common setting α=0.2; the separate sensitivity analysis in Table 4 reports the full α sensitivity for each backbone. Table 3 compares B2M with representative imbalance-aware baselines across four CNN backbones. Overall, loss re-weighting methods (Focal Loss and CB Loss) yield lower performance, remaining below 48% balanced accuracy on most backbones, which indicates that adjusting loss weights alone has limited impact under severe class imbalance. Manifold-MixUp [19] interpolates features randomly without considering class skew, resulting in lower metrics and higher empirical spread (e.g., a median balanced accuracy of 49.18% and a Q1–Q3 interval of [1.65, 4.20] on ResNeXt-50). Balanced-MixUp [17] addresses distribution skew and applies mixing at the image level, achieving a balanced accuracy of 50.45% [1.50, 3.85] on ResNeXt-50. In contrast, B2M decouples these mechanics: Phase I (dual sampling) pretrains the feature space, while Phase II feature mixing introduces a soft-target regularization that forces predictions on $z^{\prime }$ to remain independently anchored to both original labels ($y_{\mathrm{inst}}$ and $y_{\mathrm{cls}}$) simultaneously. This strategy allows B2M to achieve the best performance on ResNeXt-50, with a median balanced accuracy of 62.35% [0.55, 1.50].

This framework leads to improvements across several backbone and metric combinations. For MobileNet-V2, Class-sampling yields a slightly higher median balanced accuracy than B2M (56.30% vs. 55.72%) but exhibits a much wider IQR variance window ([1.15, 2.90] vs. [0.35, 0.95]), while B2M improves the central Macro-F1 score to 55.19%. For VGG-19, B2M improves both balanced accuracy (51.56%) and Macro-F1 (52.94%) compared to all other baselines. For DenseNet-121, Balanced-MixUp achieves the highest Macro-F1 (59.21%), yet B2M improves balanced accuracy to 56.66% with tighter overall consistency (IQR of [0.40, 1.10] vs. [1.15, 2.95]). The largest improvement occurs with ResNeXt-50, where B2M reaches 62.35% balanced accuracy and 61.87% Macro-F1.

### 5.2. Ablation Studies

#### 5.2.1. Hyperparameter Sensitivity: α

The interpolation coefficient α controls the strength of latent feature mixing and therefore determines the amount of regularization applied during training (See in Table 4). The best value of α depends on the backbone rather than following a single universal setting. For B2M, MobileNet-V2 achieves its best performance at α=0.6 (59.41% balanced accuracy with a tight [0.30, 0.85] IQR and 59.37% Macro-F1). VGG-19 performs best at α=0.3 (55.53% balanced accuracy and 54.44% Macro-F1).

For the deeper backbones, the best setting uses weaker mixing. DenseNet-121 achieves its best balanced accuracy and Macro-F1 at α=0.1 (59.98% balanced accuracy and 57.34% Macro-F1), while ResNeXt-50 achieves its best performance at α=0.2 (62.35% balanced accuracy and 61.87% Macro-F1). When α becomes too large, performance generally decreases. For example, DenseNet-121 drops from 59.98% balanced accuracy at α=0.1 to 53.45% at α=0.4. These results indicate that α should be selected according to model capacity and backbone behavior, with stronger mixing suitable for some lightweight models and more conservative mixing preferable for deeper networks.

#### 5.2.2. Layer Selection

To evaluate the dynamic layer-selection policy, we compared uniformly sampled feature mixing with fixed-depth feature mixing using the ResNeXt-50 backbone (α=0.2), as shown in Table 5. All configurations used the same Phase I dual-sampling initialization and the same soft-target regularization objective, so the layer-selection strategy was the only variable. The results show that constraining feature mixing to a single block reduced performance. Among the fixed-depth settings, the upper-mid block (layer3) performed best, achieving 58.42% median balanced accuracy and 57.90% Macro-F1. This suggests that using only one mixing depth may limit the range of learned feature abstractions. B2M addresses this limitation by uniformly sampling the hidden mixing layer from the mid-to-late stages {2,3,4} for each batch. This strategy achieved 62.35% balanced accuracy and 61.87% Macro-F1 in this setting. Varying the interpolation depth may reduce dependence on a single abstraction scale and support a more flexible latent representation for transferring benign patterns to malignant categories.

#### 5.2.3. Training Strategy

To assess the contribution of the two-phase optimization scheme, we ablated the training strategy using the ResNeXt-50 backbone (α=0.2), as shown in Table 6. The proposed approach was compared with a single-phase baseline in which dual sampling and feature mixing were applied together from the start of training. The single-phase configuration achieved 54.10% balanced accuracy and 53.65% Macro-F1. Introducing feature mixing before the encoder has learned stable class-specific representations may provide less reliable semantic supervision and weaken decision-boundary formation. In contrast, the proposed two-phase strategy first trains the model with dual sampling to stabilize the latent representation space. The model is then fine-tuned with dual sampling and feature-space mixing. This sequential design gives the encoder an initial representation before interpolation is introduced, which may help mixed features cover underrepresented regions of the feature space. The two-phase strategy achieved 62.35% balanced accuracy and 61.87% Macro-F1. These results suggest that an initial dual-sampling phase is useful for feature-level oversampling under class imbalance.

#### 5.2.4. Quantifying Benign-Share Benefit

Table 7 isolates the contribution of B2M by comparing performance before and after Phase II activation. In Phase I, the model is trained using dual sampling only, which combines instance-based and class-balanced sampling to expose the network to both natural data variation and minority-class examples. In Phase II, B2M adds feature-space mixing and soft-target regularization to the Phase I model. This comparison evaluates the benefit of feature mixing after the representation has been initialized by balanced sampling. Performance improved for every backbone after B2M activation. The largest absolute improvement was observed for ResNeXt-50, for which balanced accuracy increased from 46.95% to 62.35% (+15.40 percentage points) and Macro-F1 increased from 47.60% to 61.87% (+14.27 percentage points). VGG-19 and MobileNet-V2 also showed substantial balanced-accuracy gains (+11.63 and +10.86 percentage points, respectively), indicating that feature-space mixing provides additional benefit beyond dual sampling alone.

### 5.3. Additional Clinical Evaluation

We further evaluated the clinical behavior of the selected configuration, ResNeXt-50 with α=0.2, by comparing dual sampling (Phase I) with B2M (Phase II) (Section 5.2.4). This subsection reports three complementary analyses, including confusion matrices for BI-RADS category-level errors alongside ROC and precision–recall curves for threshold-based discrimination.

#### 5.3.1. Confusion Matrix Analysis

**Figure 6:**
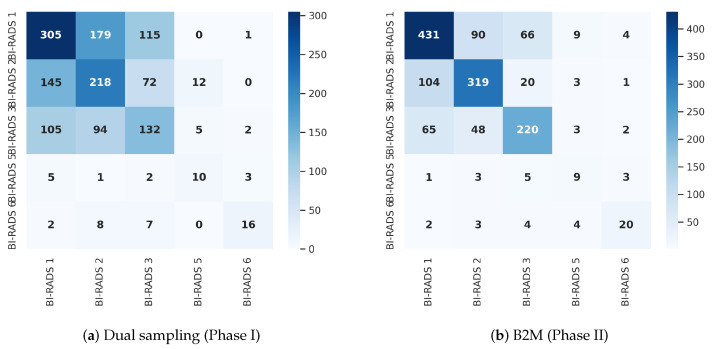
Confusion matrices for (**a**) dual sampling (Phase I) and (**b**) B2M (Phase II).

Figure 6 compares the confusion matrices for dual sampling and B2M. Compared with dual sampling, B2M shows more predictions along the main diagonal, suggesting fewer category-level errors across BI-RADS classes. This pattern is clinically relevant because fewer false negatives may reduce missed high-risk cases, and fewer false positives may reduce unnecessary recalls and follow-up examinations. However, some cases remain difficult to classify for both B2M and the alternative methods reported in Section 5.1, indicating that further improvement is still needed.

#### 5.3.2. ROC and Precision–Recall Curve Analysis

**Figure 7:**
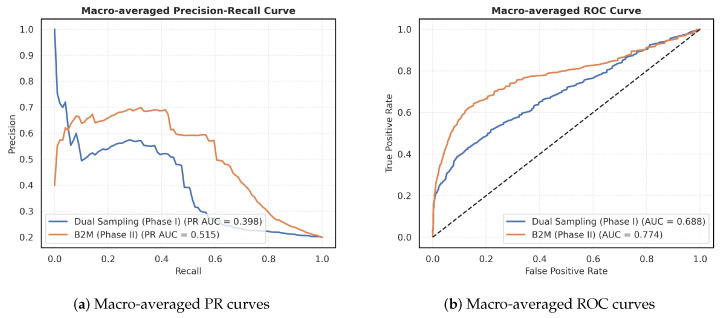
Performance comparison using (**a**) macro-averaged precision–recall (PR) curves and (**b**) macro-averaged receiver operating characteristic (ROC) curves.

Figure 7 shows the macro-averaged precision–recall (PR) and receiver operating characteristic (ROC) curves for dual sampling and B2M. The macro PR AUC increased from 39.80% with dual sampling to 51.50% with B2M (Figure 7a), while the macro ROC AUC increased from 68.80% to 77.40% (Figure 7b). These results indicate that B2M improves discrimination across decision thresholds. The PR AUC improvement is particularly relevant for breast cancer screening because PR curves are sensitive to performance on underrepresented high-risk BI-RADS categories.

#### 5.3.3. More Quantitative Metrics

Table 8 summarizes the global clinical metrics. Balanced accuracy and Macro-F1 describe overall class-balanced performance. Macro specificity measures false-positive control. Macro positive predictive value (Macro PPV) reflects the reliability of positive predictions, and macro negative predictive value (Macro NPV) reflects the reliability of low-risk predictions. ROC AUC and PR AUC evaluate discrimination across decision thresholds. PR AUC is especially relevant in this setting because high-risk BI-RADS categories are less frequent than low-risk categories. Compared with dual sampling, B2M improved all reported global metrics. Balanced accuracy increased from 46.95% to 62.35%, Macro-F1 from 47.60% to 61.87%, macro specificity from 84.30% to 90.86%, Macro PPV from 49.58% to 61.81%, and Macro NPV from 84.29% to 90.88%. These results suggest better class-balanced recognition and more reliable positive and low-risk predictions.

The per-class results in Table 9 indicate that B2M improved sensitivity for BI-RADS 1, 2, 3, and 6, reaching 71.83%, 71.36%, 65.09%, and 60.61%, respectively. Sensitivity for BI-RADS 5 decreased from 47.62% to 42.86%. This result should be interpreted cautiously because the BI-RADS 5 subgroup contains only 21 cases, so one misclassified case changes the percentage by approximately 4.76%. Specificity remained high for malignant categories under B2M, reaching 98.66% for BI-RADS 5 and 99.29% for BI-RADS 6. Overall, these findings suggest that B2M improves class-balanced mammography classification while maintaining high specificity for high-risk categories.

### 5.4. Limitations

Although B2M improves class-imbalanced mammography classification and can be integrated with standard CNN backbones without changing the inference workflow, several limitations should be considered before clinical deployment:

- **Training cost:** B2M increases offline training time by approximately 2.81× per epoch and peak GPU memory by approximately 10% on an NVIDIA GeForce RTX 2080 Ti. This does not affect inference, but it may slow model tuning and hyperparameter search.
- **Limited external validation:** The experiments were conducted on a retrospective C-view mammography cohort from EMBED. Before clinical use, further validation is needed on external datasets from multiple hospitals, scanners, acquisition protocols, and patient populations to assess robustness, calibration, and generalizability in real screening environments.
- **Limited explainability:** B2M performs interpolation in latent feature space rather than image space, making visual interpretation more difficult. Additional explainability analysis and clinical evaluation, such as reader studies or prospective testing, are needed before clinical use.

## 6. Conclusions and Future Design

### 6.1. Conclusions

This study introduced B2M (Benign Share Benefit to Malignant), a feature-space imbalance-learning strategy for multi-class BI-RADS classification in C-View mammography. B2M combines dual-stream sampling, feature-space interpolation, and soft-target regularization to address the skewed distribution across BI-RADS categories in the EMBED C-View cohort. Experiments across multiple CNN backbones showed consistent improvements, with the largest gain observed using ResNeXt-50. Because B2M is applied only during training, it keeps the inference architecture unchanged and does not add deployment-time computational overhead. However, its generalizability and clinical usefulness require further evaluation through multi-center external validation, explainability studies, and prospective testing before use in safety-critical screening workflows.

### 6.2. Future Design

Future work will focus on two directions. First, we will integrate attention-guided feature selection [3,18] to constrain interpolation around diagnostically salient structures. Second, we will extend B2M to privacy-preserving collaborative settings, including distributed and federated training across institutions [4].

## Figures and Tables

**Table 1:** Comparison of representative imbalance-learning methods and the proposed B2M framework.

Method	Mixing Space	Loss Regularization	Dual Sampling	Two-Training Strategy	Overhead
Focal Loss [13]	None	Cost-Sensitive	No	No	Low
Class-balanced [34]	None	Cost-Sensitive	No	No	Low
LDAM Loss [35]	None	Margin-Based	No	No	Low
Oversampling [40]	Input-level	None	No	No	Low
SMOTE [42]	Input-level	None	Yes	No	Low
Prior GAN [11]	Input-level	Adversarial	No	No	High
SNGAN [12]	Input-level	Adversarial	No	No	High
Balanced-MixUp [17]	Input-level	Soft-Target	Yes	No	Low
CMO [16]	Input-level	Soft-Target	No	No	Low
**B2M (Ours)**	**Feature-level**	**Soft-Target**	**Yes**	**Yes**	**High**

**Table 2:** Hyperparameters for our proposed B2M.

Pipeline Stage	Hyperparameter	Value/Setting
Preprocessing & Input	Image Target Resolution	512×512 pixels
	Min–Max Normalization Range	[0,255]
	Segmentation Binarization Threshold	50
	Bounding Box Square Expansion Factor	1.2×
	CLAHE Clip Limit	2.0
	CLAHE Local Tile Grid	8×8
Augmentation	Rotation Range	Up to $\pm 15^{\circ }$
	Scaling Factor	1.2×
	Horizontal Translation	5%
	Brightness and Contrast Range	±10%
	Horizontal Flip	Probability = 0.5
Optimization	Optimizer	SGD
	Initial Learning Rate (η)	0.01
	Momentum	0.9
	Weight Decay	$1\times 10^{-4}$
	LR Schedule	Cosine Annealing
	Batch Size	8
Phase I (Dual Sampling)	Power Sampling Exponents (*q*)	Dual streams active: q=0 and q=1
	Early Stopping Patience (*n*)	10 epochs
	Early Stopping Threshold (ϵ)	$10^{-4}$
	Maximum Training Limit	50 epochs
Phase II (B2M Fine-tuning)	Feature Mixup Parameter (α)	Backbone-specific; see Table 4
	Candidate Hidden Layers (S)	Uniformly sampled from mid-to-late stages {2, 3, 4}
	Total Fine-tuning Epochs ($E_{\mathrm{fine}}$)	40 epochs
Reproducibility	Global Random Seed	42 (Fixed across folds)

**Table 3:** Comparative balanced accuracy (%) and Macro-F1 (%) across four CNN backbones at α=0.2. Results are reported as median [Q1, Q3] across five cross-validation folds. The best result for each backbone and metric is shown in **bold**.

**Method**	**MobileNet-V2**		**VGG-19**	
	**Balanced Accuracy**	**Macro-F1**	**Balanced Accuracy**	**Macro-F1**
Class-sampling	**56.30** [1.15, 2.90]	53.10 [1.25, 3.20]	50.48 [0.90, 2.35]	50.71 [1.05, 2.70]
Focal Loss [13]	45.76 [0.85, 2.20]	44.04 [0.80, 2.10]	45.74 [0.95, 2.45]	43.78 [0.85, 2.20]
CB Loss [34]	46.88 [0.70, 1.90]	45.35 [0.90, 2.30]	45.68 [0.80, 2.05]	43.51 [0.75, 1.90]
Manifold-MixUp [19]	49.35 [1.25, 3.25]	50.14 [1.40, 3.55]	48.70 [1.15, 2.90]	49.02 [1.20, 3.05]
Balanced-MixUp [17]	50.89 [1.05, 2.75]	52.19 [1.20, 3.10]	50.16 [1.00, 2.60]	50.87 [1.10, 2.80]
**B2M (ours)**	55.72 [0.35, 0.95]	**55.19** [0.40, 1.05]	**51.56** [0.30, 0.85]	**52.94** [0.45, 1.20]
**Method**	**DenseNet-121**		**ResNeXt-50**	
	Balanced accuracy	Macro-F1	Balanced accuracy	Macro-F1
Class-sampling	55.42 [0.95, 2.45]	53.48 [1.15, 3.00]	51.65 [1.45, 3.75]	51.26 [1.35, 3.50]
Focal Loss [13]	46.08 [0.75, 1.95]	45.58 [0.80, 2.05]	45.35 [1.10, 2.80]	43.57 [0.95, 2.50]
CB Loss [34]	47.03 [0.90, 2.30]	45.58 [0.95, 2.40]	47.35 [1.00, 2.65]	47.30 [1.15, 2.90]
Manifold-MixUp [19]	53.12 [1.35, 3.50]	54.85 [1.45, 3.80]	49.18 [1.65, 4.20]	48.90 [1.50, 3.90]
Balanced-MixUp [17]	55.69 [1.15, 2.95]	**59.21** [1.30, 3.30]	50.45 [1.50, 3.85]	50.18 [1.40, 3.60]
**B2M (ours)**	**56.66** [0.40, 1.10]	56.31 [0.50, 1.35]	**62.35** [0.55, 1.50]	**61.87** [0.65, 1.70]

**Table 4:** Sensitivity analysis of the interpolation coefficient α using balanced accuracy (%) and Macro-F1 (%). Results are reported as median [Q1, Q3] across folds, and the best result within each method block, backbone, and metric is highlighted in **bold**.

α	**MobileNet-V2**		**VGG-19**	
	**Balanced Accuracy**	**Macro-F1**	**Balanced Accuracy**	**Macro-F1**
*Balanced-MixUp [17]*				
0.1	48.77 [1.15, 3.00]	48.93 [1.10, 2.95]	47.32 [1.00, 2.65]	46.58 [0.95, 2.60]
0.2	50.89 [1.05, 2.75]	52.19 [1.20, 3.10]	50.16 [1.00, 2.60]	50.87 [1.10, 2.80]
0.3	50.43 [1.10, 2.85]	51.14 [1.05, 2.80]	43.90 [1.25, 3.25]	41.40 [1.20, 3.10]
0.4	**52.93** [0.95, 2.45]	53.25 [1.00, 2.60]	47.38 [1.10, 2.90]	47.09 [1.05, 2.75]
0.5	52.36 [1.15, 3.00]	**54.37** [1.20, 3.15]	**50.93** [0.90, 2.40]	**52.70** [0.95, 2.50]
0.6	48.55 [1.25, 3.20]	48.87 [1.25, 3.25]	46.74 [1.15, 3.00]	46.59 [1.10, 2.95]
*Proposed: B2M (ours)*				
0.1	57.05 [0.40, 1.05]	56.49 [0.45, 1.20]	50.84 [0.35, 1.00]	49.91 [0.40, 1.10]
0.2	55.72 [0.35, 0.95]	55.19 [0.40, 1.05]	51.56 [0.30, 0.85]	52.94 [0.45, 1.20]
0.3	57.42 [0.45, 1.20]	56.56 [0.40, 1.00]	**55.53** [0.30, 0.75]	**54.44** [0.35, 0.90]
0.4	54.54 [0.45, 1.25]	52.76 [0.45, 1.15]	52.64 [0.40, 1.00]	53.32 [0.35, 1.00]
0.5	53.64 [0.40, 1.10]	52.11 [0.50, 1.30]	47.91 [0.45, 1.15]	48.09 [0.45, 1.20]
0.6	**59.41** [0.30, 0.85]	**59.37** [0.35, 0.95]	51.33 [0.35, 0.90]	51.66 [0.40, 1.05]
α	**DenseNet-121**		**ResNeXt-50**	
	Balanced accuracy	Macro-F1	Balanced accuracy	Macro-F1
*Balanced-MixUp [17]*				
0.1	**56.56** [1.05, 2.80]	57.12 [1.10, 2.90]	47.96 [1.35, 3.50]	47.36 [1.40, 3.65]
0.2	55.69 [1.15, 2.95]	**59.21** [1.30, 3.30]	50.45 [1.50, 3.85]	50.18 [1.40, 3.60]
0.3	55.00 [1.15, 3.00]	56.45 [1.20, 3.15]	51.10 [1.30, 3.35]	50.30 [1.35, 3.45]
0.4	55.29 [1.05, 2.70]	55.18 [1.10, 2.80]	49.45 [1.40, 3.60]	48.86 [1.40, 3.60]
0.5	51.94 [1.25, 3.20]	53.69 [1.30, 3.40]	53.69 [1.25, 3.30]	53.12 [1.30, 3.40]
0.6	43.57 [1.40, 3.65]	45.19 [1.45, 3.80]	**54.19** [1.45, 3.70]	**54.87** [1.45, 3.80]
*Proposed: B2M (ours)*				
0.1	**59.98** [0.45, 1.20]	**57.34** [0.40, 1.10]	59.44 [0.65, 1.60]	58.90 [0.60, 1.60]
0.2	56.66 [0.40, 1.10]	56.31 [0.50, 1.35]	**62.35** [0.55, 1.50]	**61.87** [0.65, 1.70]
0.3	54.12 [0.50, 1.30]	53.94 [0.55, 1.40]	60.18 [0.60, 1.60]	59.62 [0.65, 1.65]
0.4	53.45 [0.45, 1.15]	52.76 [0.50, 1.25]	58.70 [0.70, 1.75]	57.95 [0.60, 1.60]
0.5	51.61 [0.55, 1.40]	50.88 [0.50, 1.35]	56.43 [0.70, 1.80]	55.13 [0.75, 1.85]
0.6	53.20 [0.40, 1.05]	52.10 [0.45, 1.20]	60.68 [0.55, 1.40]	60.81 [0.60, 1.50]

**Table 5:** Layer-selection ablation using ResNeXt-50 (α=0.2). Results are reported as median [Q1, Q3] across folds.

Layer-Selection Strategy (Phase II)	Balanced Accuracy (%)	Macro-F1 (%)
Fixed lower-mid block (layer2)	55.34 [0.85, 2.25]	54.82 [0.90, 2.35]
Fixed upper-mid block (layer3)	58.42 [0.65, 1.75]	57.90 [0.70, 1.85]
Fixed deep block (layer4)	56.80 [0.70, 1.90]	55.45 [0.75, 2.05]
**Uniformly sampled from** {2,3,4} **(B2M)**	**62.35** [0.55, 1.50]	**61.87** [0.65, 1.70]

**Table 6:** Training-strategy ablation using ResNeXt-50 (α=0.2). Results are reported as median [Q1, Q3] across folds.

Training Strategy	Balanced Accuracy (%)	Macro-F1 (%)
Single-phase training	54.10 [0.90, 2.45]	53.65 [1.00, 2.60]
**Two-phase training (ours)**	**62.35** [0.55, 1.50]	**61.87** [0.65, 1.70]

**Table 7:** Phase-wise ablation study comparing the baseline using dual sampling only (Phase I) against the model including B2M fine-tuning (Phase II). Results are reported as median [Q1, Q3] across folds. The best result for each backbone and metric is shown in **bold**.

Model (α)	Dual sampling (Phase I)		B2M (Phase II)	
	Balanced Accuracy	Macro-F1	Balanced Accuracy	Macro-F1
MobileNet-V2 (0.6)	48.55 [1.25, 3.20]	48.87 [1.25, 3.25]	**59.41** [0.30, 0.85]	**59.37** [0.35, 0.95]
VGG-19 (0.3)	43.90 [1.25, 3.25]	41.40 [1.20, 3.10]	**55.53** [0.30, 0.75]	**54.44** [0.35, 0.90]
DenseNet-121 (0.2)	53.16 [1.00, 2.70]	55.49 [1.05, 2.85]	**56.66** [0.40, 1.10]	**56.31** [0.50, 1.35]
ResNeXt-50 (0.2)	46.95 [1.35, 3.55]	47.60 [1.25, 3.30]	**62.35** [0.55, 1.50]	**61.87** [0.65, 1.70]

**Table 8:** Global clinical metrics for dual sampling and B2M using ResNeXt-50 (α=0.2).

Metric	Dual Sampling	B2M
Balanced accuracy (%)	46.95	**62.35**
Macro-F1 (%)	47.60	**61.87**
Macro specificity (%)	84.30	**90.86**
Macro PPV (%)	49.58	**61.81**
Macro NPV (%)	84.29	**90.88**
ROC AUC (%)	68.80	**77.40**
PR AUC (%)	39.80	**51.50**

**Table 9:** Per-class sensitivity and specificity for dual sampling and B2M using ResNeXt-50 (α=0.2).

BI-RADS	*N*	Sensitivity (%)		Specificity (%)	
		Dual Sampling	B2M	Dual Sampling	B2M
1	600	50.83	**71.83**	69.37	**79.50**
2	447	48.77	**71.36**	71.57	**85.48**
3	338	39.05	**65.09**	82.20	**91.37**
5	21	**47.62**	42.86	**98.80**	98.66
6	33	48.48	**60.61**	**99.57**	99.29

## Data Availability

The Emory Breast Imaging Dataset (EMBED) used in this study is publicly available in the Registry of Open Data on AWS at https://registry.opendata.aws/emory-breast-imaging-dataset-embed/ (accessed on 1 January 2024) [29]. Because EMBED is a publicly available, fully de-identified clinical repository, this study constitutes non-human subjects research. The data were obtained retrospectively from public records, and no new patient consent or direct clinical data collection was required or performed by the authors.
